# Case report of a large lipoma discovered intraoperatively in a chronically irritated implantable cardioverter-defibrillator pocket

**DOI:** 10.1093/ehjcr/ytac245

**Published:** 2022-06-21

**Authors:** Heiko Burger, Gerhard Göbel, Manfred Richter, Simon Pecha

**Affiliations:** Department of Cardiovascular Surgery, Heart Center Kerckhoff-Klinik, 61231 Bad Nauheim, Germany; Campus Kerckhoff, Justus-Liebig-University Giessen, 61231 Bad Nauheim, Germany; Department of Angiology and Cardiology, CardioVascular Center Frankfurt (CVC), 60389 Frankfurt, Germany; Department of Cardiovascular Surgery, Heart Center Kerckhoff-Klinik, 61231 Bad Nauheim, Germany; Campus Kerckhoff, Justus-Liebig-University Giessen, 61231 Bad Nauheim, Germany; Department of Cardiovascular Surgery, Heart Center Kerckhoff-Klinik, 61231 Bad Nauheim, Germany; Campus Kerckhoff, Justus-Liebig-University Giessen, 61231 Bad Nauheim, Germany; Department of Cardiovascular Surgery, University Heart Center, 20251 Hamburg, Germany

**Keywords:** Case report, Lipoma, Pocket, Pacemaker, ICD, CIED, Complication

## Abstract

**Background:**

The implantation of cardiac implantable electronic devices is a globally established therapy to treat cardiac arrhythmias with low complication rates. Apart from technical problems, however, complications can arise from the implanted material. This can lead to bleeding, infections, or chronic irritation of the generator pocket, resulting in swellings, seromas, perforations, or fistulas. However, the cause of tissue changes is not always clear, and therefore, we would like to report on a rare tissue degeneration diagnosed in a patient.

**Case summary:**

After a history of ventricular fibrillation, a 46-year-old patient received an implantable cardioverter-defibrillator (ICD) for secondary prevention. Six years later, the generator pocket swelled without evidence of infection. With the suspected diagnosis of a chronically irritated pocket, the device was then surgically relocated. After a 2-year symptom-free period, the patient presented again with a severely swollen but only slightly painful device pocket. Once again, there were no signs of infection, and so the pocket was revised again, assuming a chronic irritant effusion. Intraoperatively, a lipomatous structure (12 × 6 × 3 cm) emerged from the subpectoral ICD pocket. After its complete removal, the histopathological examination revealed a lipoma. A bacterial genesis could be ruled out by microbiological samples, and the wound healed cosmetically well and without further discomfort.

**Conclusion:**

This case shows that the reason of chronically irritated generator pockets, in addition to the usually known tissue changes, can also be tumours. Therefore, resected tissue should be examined histopathologically and, if indicated, specific therapy initiated.

**Learning points**

- ­ Unusual changes in device pockets can also rarely be mesenchymal tumour.
- ­ Suspicious tissue should therefore be removed and histopathologically examined in order to differentiate malignant soft tissue tumours such as liposarcoma from benign tumours such as lipomas and, if necessary, to initiate targeted therapy in good time.

## Introduction

Based on international guidelines,^1,2^ the implantation of cardiac pacemakers (PM) and implantable cardioverter-defibrillator (ICD) has become an integral part in clinical practice treating bradycardic and tachycardiac arrhythmias. Despite low complication rates during de novo implantations (e.g. pneumothorax 0.8%, lead dislocation 0.8%, pericardial effusion 0.2%, pocket haematoma 0.1%, or resuscitation measures less than 0.2%), the annual revision rate is 10.3% for PMs and 19.8% for ICDs in relation to all annual device interventions performed in Germany 2020.^3,4^ Among these, 33.6% (PM) and 41.9% (ICD) represent aseptic complications, which can be divided into technical problems or mechanical pocket irritations with swelling or pain. Furthermore, 10.7% (ICD) respectively 12% (PM) of revision procedures are due to bacterial infections. These can be the result of system perforations or wound healing disorders, which pose a high risk of developing life-threatening endocarditis.^3–5^

While acutely infected generator pockets are usually identified quickly, chronic wound healing disorders or deep tissue irritations with non-specific symptoms or missing evidence of infection are sometimes difficult to verify. The lack of diagnosis then often delays targeted therapy, and therefore, we would like to present an unusual case of a very rare wound reaction after ICD implantation.

## Timeline

**Table:** 

Timeline (patient's age)	Measures and findings
0	Congenital pulmonary atresia with ventricular septal defect (VSD).
…	Several corrective cardiac surgeries were performed to treat congenital heart defects.
29	Final corrective cardiac surgery for VSD closure and pulmonary valve reconstruction.
31	Catheter-assisted atrial ablation due to symptomatic supraventricular tachycardia.
35	Catheter-assisted atrial ablation for recurrence of supraventricular tachycardia.
38	ICD (Vitality VR, Guidant) implantation for secondary prevention after sudden cardiac arrest due to ventricular fibrillation.
38	Surgical pocket revision due to a postoperative pocket haematoma.
39	HF ablation of an identified arrhythmogenic right ventricular substrate.
43	Re-HF ablation of an identified arrhythmogenic right ventricular substrate.
43	ICD generator exchange (Marquis VR, Medtronic) due to battery depletion.
44	Surgical pocket revision due to an aseptic painfully swollen generator pocket.
44	Postoperative pocket haematoma not requiring treatment.
46	Another surgical pocket revision due to a severely swollen but only slightly painful generator pocket
46	Histopathological evidence of a lipoma and microbiological exclusion of a bacterial genesis.
…	After lipoma resection, there have been no further complaints to date.

## Background

We report on a male patient (176 cm/77 kg) who experienced multiple operations as a child for congenital pulmonary atresia with ventricular septal defect (VSD) and finally underwent VSD closure and pulmonary valve reconstruction at the age of 29 years. Furthermore, two catheter-supported atrial ablations were performed at the age of 31 and 35 due to symptomatic supraventricular tachycardia. In addition, at the age of 38 he had to be resuscitated for ventricular fibrillation and received a secondary prophylactic ICD (Vitality VR, Guidant) according to the guidelines.^1,2^ Unfortunately, due to a progressive pocket haematoma, surgical revision became necessary. However, there were no further postoperative complications and the patient was able to leave the hospital three days thereafter in good condition.

At the age of 43, an ICD generator exchange (Marquis VR, Medtronic) had to be performed due to low battery capacity. This was followed by a complication-free year without any cardiac arrhythmia and flawless device function before the athletic patient presented to our hospital with a painfully swollen generator pocket. Interestingly, no signs of infection such as fever, chills, local redness, swelling, or pain could be detected. Blood samples taken also depicted unremarkable infection parameters such as leucocytes, C-reactive protein (CRP), and procalcitonin (PCT), and the blood cultures additionally taken showed no germs. Moreover, a comprehensive examination of the generator pocket illustrated no signs of infection, and an ultrasound scan showed no evidence of free fluid. Also, no vegetation on cardiac structures or electrodes and no intracardiac thrombi were seen. Therefore, the most likely cause of his complaints seemed to be an ICD dislocation due to mechanical influences. Subsequent pocket revision confirmed this, and thickened taut scar tissue around the device was removed and the generator repositioned. Unfortunately, a small pocket haematoma occurred but did not require surgical treatment. So, he was able to leave the hospital 4 days later without any further complaints and unchanged medication (valsartan 80 mg, acetylsalicylic acid 100 mg, bisoprolol 2.5 mg).

**Figure 1:**
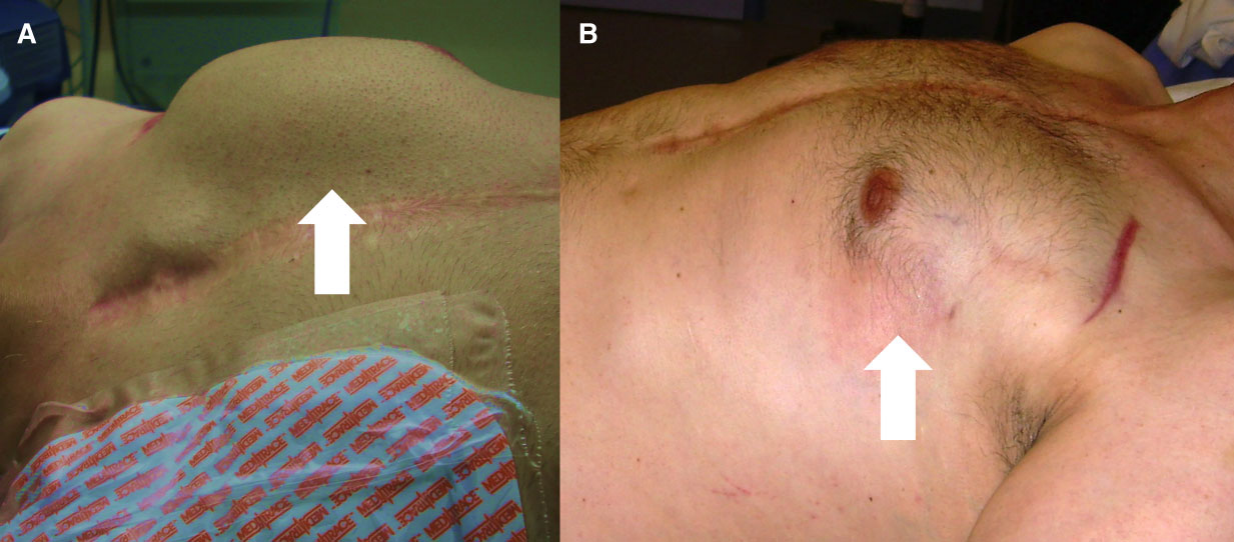
External appearance of the device pocket before (*A*) and after surgical revision (*B*). (*A*) Massively swollen generator pocket (white arrow) in lateral view. (*B*) Generator pocket (white arrow) 3 months after lipoma resection.

**Figure 2:**
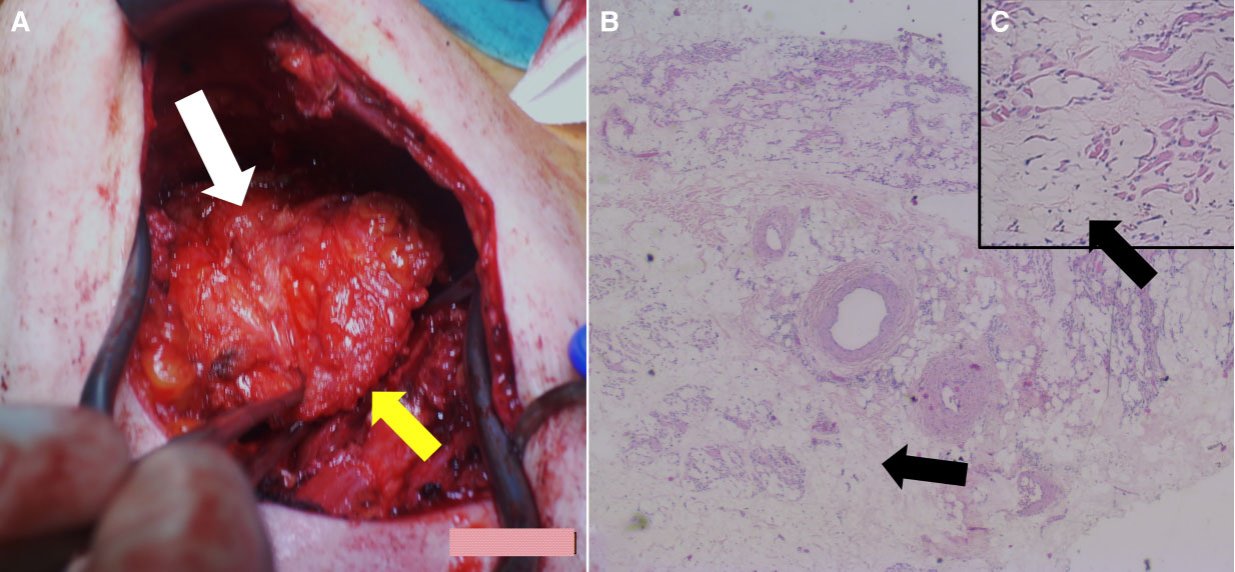
Presentation of the intraoperative lipoma findings (*A*) and the histological image (*B/C*), (*A*) Intraoperative finding of the large lipoma (white arrow above), which arises from the subpectoral generator pocket (pocket entrance is marked with yellow arrow bottom right ). (*B/C*) The histological tissue section shows numerous apparently mature adipocytes (black arrows), which are typical of a lipoma. This can be seen in detail in the enlarged section (*C*).

Unfortunately, after 2 symptom-free years, the sportive 46-year-old patient returned with a severely swollen but only slightly painful generator pocket (*Figure 1A/B*). Again, there were neither fever, nor increased laboratory inflammation parameters, nor local signs of inflammation found, and the most likely cause seemed once more to be a mechanical pocket irritation. This time, an ultrasound scan showed a few millimetres of free fluid in the ICD pocket, but the amount did not explain the big swelling (*Figure 1A/B*). However, the hypoechoic heterogenous structures appeared thicker compared to other generator pockets but not noticeably structurally altered. In addition, the cardiac ultrasound examination revealed again no intracardiac vegetations or thrombi, no relevant heart valve defects, and an unchanged good left ventricular ejection fraction (60%). Due to these ambiguous findings, another intervention was scheduled. Here, the subcutaneous tissue was found to be scarred, and deeper tissue dissection revealed a several centimetres dehiscent subpectoral pocket entrance giving ride to a large adipose tissue-like structure. This emerged from this muscle gap and originated in the subpectoral pocket ventral to the generator and invaded the subcutaneous tissue dorsal to the left mammilla (*Figure 2A*). For this purpose, the tissue (12 × 6×3 cm) was completely resected, the old generator pocket closed, and a new one created further medially, into which the ICD generator could then be inserted. The final results of the perioperatively taken swab cultures and tissue samples did not indicate any bacterial presence. However, the result of the histopathological tissue examination was surprising. Here, lobulated mature adipose tissue with monomorphic peripheral cell nuclei was found without evidence of lipoblasts. There were also sparse lymphoid inflammatory infiltrates and isolated flattened skeletal muscle fibres with scattered myogenic giant cells. Overall, the resection showed numerous apparently mature adipocytes in a typical arrangement that led to the diagnosis of a large benign lipoma (*Figure 2B,C*). Finally, the patient was able to leave the hospital without further complaints. For further treatment of his underlying diseases, he was recommended to continue taking the necessary medication (valsartan 80 mg, acetylsalicylic acid 100 mg, bisoprolol 2.5 mg). A wound check three months later revealed irritation-free wound conditions with good cosmetic results (*Figure 1B*). There were no further complaints during the six-monthly ICD checks that followed, and no indications of tumour recurrence 8 years after the operation.

## Discussion

The occurrence of lipomas on the trunk or extremities is an uncommon event^6–8,13^ and even rarer in chronically irritated device pockets^12,14,15^. Previous investigations have shown that lipomatous tumours at 50% represent the most common group of mesenchymal growths with a mostly good prognosis. They usually arise subcutaneously or subfascially and show a typical age peak in the 4th to 6th decade of life in 1% of the population. Surgical treatment is only indicated in case of pain, restrictions of movement, or extensive supplant of the surrounding tissue.^6–8^ The currently largest retrospective analysis (428 patients)^11^ on size, location, and occurrence of lipomas showed that 79% were solitary, subcutaneous, and smaller than 5 cm. Multiple lipomas occurred in 14% and only 3% were subfascial or deeper. The deeper ones measured over 6 cm and were thus twice the size of the subcutaneous lipomas. In addition, the risk of transformation into a liposacroma or the also mesenchymal, highly malignant cutaneous leiomyosarcoma^13,15^ increased with tumor size (> 5 cm) and tissue depth.^8^ Another smaller study^9^ was able to show that 70% of lipomas were caused by sustained tissue stress such as blunt soft tissue injuries with extensive and slowly resorbing haematomas. From these, cytokines seem to be released directly, which cause the differentiation of mature preadipocytes or indirect trigger factors arise which lead to formation of lipomas.^10^ But also, local inflammatory reactions, an increased partial thromboplastin time, or a high body mass index could promote tumourigenesis from trauma-related adipose tissue necrosis.^9^ On the other hand, a recently published case report^14^ on the occurrence of large lipomas 3.5 to 8 years after de novo ICD-implantation showed that these can occur elsewhere without surgical (re)interventions or pre-existing lipomas. A retrospective analysis of 6.400 implants with an 8-year follow-up revealed a 0.04% incidence rate for lipomatous neoplasia but no association with specific ICD manufacturers or models.^14^ However, there are also reports of cutaneous malignant leiomyosarcomas over PM pockets,^15^ that have developed after repeated switching between iron- or titanium-coated devices from different manufactures and different epochs. Similar to orthopedic implants, there is still no conclusive evidence of a direct link to tumour genesis.

In our case, analogous to other authors,^9,10^ an aseptically multifactorial genesis seems to have caused the development of the lipoma, with the relevant influences being reflected in the repeated interventions with bleeding complications and sustained mechanical tissue stress. However, since we assumed an aseptic process, we refrained from a possible positron emission tomography and computed tomography scan. The accumulation of the applied radionuclide 18F-fluorodeoxyglucose in the metabolism-intensive tissue (Warburg effect) could have given us indication evidence of an infectious focus, which was not found afterwards either. Ultimately, neither genetic predispositions nor influence of changing aggregates and manufacturers^15^ can be completely ruled out as cause for lipomagenesis. Finally, based on our findings, we assume that the lipoma developed after the first pocket revision, since we could not find any tissue changes at this time apart from extensive scar tissue, which can be well discriminated. However, it also seems possible that this intervention was only the last in a chain of noxae that initiated the development of a lipoma.

Even if more and more case reports describe individual aspects of lipoma genesis, the current scientific knowledge gaps do not allow a conclusive description of a causal chain. There is also no clear evidence to date on the efficacy of adjuvant chemotherapy or radiotherapy in malignant mesenchymal tumours.^13^ Against this background, it seems necessary to identify trigger factors for malignant conversion. Currently, the recommendation remains for the timely surgical removal of suspicious tissue structures with a sufficient safety margin. This measure significantly improves the prognosis, especially in the case of malignant neoplasms, and reduces the risk of recurrence. The removed tissue must be examined histopathologically^11^ and targeted therapy initiated in the case of detected malignant neoplasms (e.g. liposarcoma, angiolipoma, histiocytoma, fibrosarcoma, or other non-lipogenic malignant tumours).^5,8^

## Lead author biography


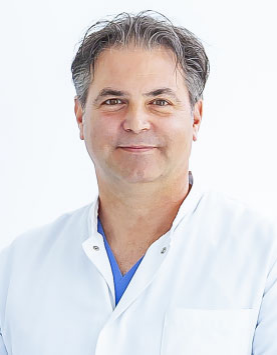
Dr. Heiko Burger studied medicine at Justus Liebig University (Giessen, Germany) and is specialist in cardiac surgery at Kerckhoff Clinic (Bad Nauheim). In 2008, he received his doctorate with basic research on endothelial nitric oxide synthesis.

## Supplementary Material

ytac245_Supplementary_Data

## Notes

**Patient consent statement:** At the time of the treatment, the patient described here consented to the anonymized scientific processing and publication of his medical history as part of the preoperative procedure documents. In addition, the patient gave his explicit consent to publication of this case report in accordance with the COPE guidelines.

**Conflict of interest:** The authors declare that there is no conflict of interest. There is no affiliation with, or interest in, any organization or entity with financial interests (eg, honoraria, educational grants, participation in voice offices, membership, employment, consulting firms, stock ownership or other interests; and expert opinion or patent license agreements). Additionally, there are no financial interests (such as personal or professional relationships, affiliations, knowledge, or beliefs) related to the topics or materials discussed in this manuscript.

**Funding:** The authors received no financial support for the research, authorship, and/or publication of this article.

## References

[1] Gregoratos G, Abrams J, Epstein AE, Freedman RA, Hayes DL, Hlatky MA, Kerber RE, Naccarelli GV, Schoenfeld MH, Silka MJ, Winters SL, Gibbons RJ, Antman EM, Alpert JS, Gregoratos G, Hiratzka LF, Faxon DP, Jacobs AK, Fuster V, Smith SC. ACC/AHA/NASPE 2002 guideline update for implantation of cardiac pacemakers and antiarrhythmia devices: summary article. Circulation 2002;106:2145–2161. 12379588 10.1161/01.cir.0000035996.46455.09

[2] Ponikowski P, Voors AA, Anker SD, Bueno H, Cleland JGF, Coats AJS, Falk V, González-Juanatey JR, Harjola V-P, Jankowska EA, Jessup M, Linde C, Nihoyannopoulos P, Parissis JT, Pieske B, Riley JP, Rosano GMC, Ruilope LM, Ruschitzka F, Rutten FH, van der Meer P. ESC guidelines for the diagnosis and treatment of acute and chronic heart failure. Eur Heart J 2016;37:2129–2200.27206819

[3] Behrens S, Burger H, Fröhlig G, Kolb C, Kollmar M, Lemke B, Macher-Heidrich S, Markewitz A, Müller WH, Noack F, Nowak B, Pätzmann-Sietas B, Stellbrink C, van Essen J, Wiegand U. Qualitätsreport 2020 zum Erfassungsjahr 2019. pp 46–65. https://iqtig.org/downloads/berichte/2019/IQTIG_Qualitaetsreport-2020_2021-02-11.pdf (22 June 2022)

[4] Annual reports 2002-2020 of the “German Pacemaker and ICD Register”. https://pacemaker-register.de/ (14 January 2022)

[5] Kusumoto FM, Schoenfeld MH, Wilkoff BL, Berul CI, Birgersdotter-Green UM, Carrillo R, Cha YM, Clancy J, Deharo JC, Ellenbogen KA, Exner D. 2017 HRS expert consensus statement on cardiovascular implantable electronic device management and extraction. Heart Rhythm 2017;14(12):e503–e551. 28919379 10.1016/j.hrthm.2017.09.001

[6] Signorini M, Campiglio GL. “Posttraumatic lipomas: where do they really come from?”. Plast Reconstr Surg 1998;101:699–705. 9500386 10.1097/00006534-199803000-00017

[7] Aust MC, Spies M, Kall S, Jokuszies A, Gohritz A, Vogt P. “Posttraumatic lipoma: fact or fiction?”. Skinmed 2007;6:266–70. 17975353 10.1111/j.1540-9740.2007.06361.x

[8] Weiss SW . Lipomatous tumors. Monogr Pathol 1996;38:207–39. 8744279

[9] Aust MC, Spies M, Kall S, Vogt PM. Diagnosis and treatment of posttraumatic pseudolipomas. A retrospective analysis. Unfallchirurg Skinmed 2006;109:948–55. 10.1007/s00113-006-1160-z 17058061

[10] Copcu E, Sivrioglu NS. Posttraumatic lipoma: analysis of 10 cases and explanation of possible mechanisms. Dermatol Surg 2003;29:215–20. 12614411 10.1046/j.1524-4725.2003.29052.x

[11] Rydholm A, Berg NO. Size, site and clinical incidence of lipoma. Acta Orthop Scand 1983;54:929–34. 6670522 10.3109/17453678308992936

[12] McCreary K, Roberts M, McKeag N. Pacemaker pocket mass: tumor within the deltopectoral groove. EP Europace 2020;22:1536. 10.1093/europace/euaa125 32533182

[13] Begum M, Hossain MS. A case report on cutaneous leiomyosarcoma with review of literature. JCAMR 2017;4:63–67.

[14] Holmes S, McEdwards G, Healey JS, Cin AD. Implantable cardioverter-defibrillator and lipomas: a case-series. Plast Surg Case Stud 2021;7:1–3.

[15] González-Vela MC, Val-Bernal JF, Rubio S, Olalla JJ, González-López MA. Cutaneous leiomyosarcoma developing on a pacemaker pocket. Dermatol Surg 2009;35:863–867. 19389092 10.1111/j.1524-4725.2009.01145.x

