# Supplementary material for: Case report of a large lipoma discovered intraoperatively in a chronically irritated implantable cardioverter-defibrillator pocket
Source: Eur Heart J Case Rep. 2022 Jun 21;6(7):ytac245. doi: 10.1093/ehjcr/ytac245 (PMC9257792; doi:10.1093/ehjcr/ytac245)
Supplement: ytac245_Supplementary_Data [file ytac245_supplementary_data.zip › 21-00918_Form for additional athors.pdf]

Request for additional authors above article limits

This form should be used by the corresponding author if they wish to request that additional authors are required above the limits outlined by the journal. Please note that submission of this form does not guarantee agreement by the editors of EHJ-Case Reports to allow additional authors to be included.

All authors should be listed and their contributions defined. All authors are required to meet the 'authorship criteria' to be considered appropriate for inclusion. A reason for requesting more authors than than the authorship limit is required and should be selected from the dropdown list, please note the requirement to provide further details for these selections

**Article Title**	Case report of a large lipoma discovered intraoperatively in a chronically irritated ICD pocket
**Article reference (if available)**	
**Article Type**	Case Report — Author Limit: 4
**Corresponding Author name**	Heiko Burger
**Full author list (in the order you would like them to appear)**	Heiko Burger, Gerhard Göbel, Manfred Richter, Simon Pecha
**Reason for requesting additional authors**	Case complexity required involvement of additional authors from other specialties (please document in notes section beside each author)
**Additional details**	Support for supplementing information and in stylistic revision of the article

Order	Author name	Author contribution: Involvement with patient care	Manuscript preparation	Final approval	Accountability	Meets authorship criteria	Notes	Author signature
1	Heiko Burger	Undertook patient investigations	Drafting manuscript	Final approval of the manuscript	Agreement to be accountable for all aspects of the work	YES		
2	Gerhard Göbel	Patient Care - Undertook	Critical revision of the manuscript	Final approval of the manuscript	Agreement to be accountable for all aspects of the work	YES		
3	Manfred Richter	Patient Care - Supervised	Critical revision of the manuscript	Final approval of the manuscript	Agreement to be accountable for all aspects of the work	YES		
4	Simon Pecha	Patient Care - Supervised	Critical revision of the manuscript	Final approval of the manuscript	Agreement to be accountable for all aspects of the work	YES		
